# Cytochrome P450 CYP 2C19*2 Associated with Adverse 1-Year Cardiovascular Events in Patients with Acute Coronary Syndrome

**DOI:** 10.1371/journal.pone.0132561

**Published:** 2015-07-06

**Authors:** You-quan Wei, Dian-gang Wang, Hao Yang, Heng Cao

**Affiliations:** 1 Department of Cardiology, The First Affiliated Hospital of Wannan Medical College, Wuhu, China; 2 Department of Cardiology, The Second People's Hospital of Huangchuan County, Xinyang, China; University of Bologna, ITALY

## Abstract

**Background:**

The cytochrome P450 (CYP450) 2C19 681 genotypes affect the antiplatelet activity of clopidogrel. We investigated the correlation of CYP 2C19 681G > A mutation with clopidogrel resistance (CR). Additionally, we studied the effect of CR on clinical prognosis of patients with acute coronary syndrome (ACS).

**Methods:**

One hundred ten ACS patients undergoing percutaneous coronary intervention, who were followed-up for 1 year, were included in the study. The patients were co-administered aspirin 100 mg/d and clopidogrel 75mg/d following a loading dose of 300 mg. CR was assessed on the basis of polymorphism observed in the CYP2C19 subgroup.

**Results:**

Patients in GG genotype group exhibited greater inhibition of platelet aggregation than patients in GA and AA genotype groups (16.2 ± 10.1%; 10.2 ± 9.9%; 8.0 ± 5.9%, respectively, *p* < 0.01). CYP2C19 681GG genotype group was associated with lower CR than CYP2C19 681A allele (GA + AA) group (9/59 vs. (12+5)/51; *p* = 0.009). Over a follow-up of 12 months, the incidence of recurrent angina, acute myocardial infarction, and intra-stent thrombosis in CYP2C19 681 GG carriers was significantly lower than that in CYP2C19 681A allele (GA + AA) group (2/59 vs. 8/51, 1/59 vs. 6/51, 0 vs. 4/51, respectively, *p* < 0.05).

**Conclusion:**

CYP 2C19*2 is associated with reduced clopidogrel antiplatelet activity and might be an important marker for poor prognosis of ACS.

## Introduction

Clopidogrel, which irreversibly inhibits the adenosine diphosphate (ADP) platelet receptor P2Y12, has been widely used in preventing thromboembolic events in patients with acute coronary syndrome (ACS). However, a great number of patients under the treatment of clopidogrel still experience cardiovascular events. Various studies have demonstrated that the rate of clopidogrel resistance (CR) ranges 4.2–27.8% [1,2]. Clopidogrel is a prodrug, which requires CYP biotransformation to convert into its active metabolite [3]. The CYP2C19 enzyme is generally known to play a vital rule in clopidogrel transformation. Among the several polymorphisms investigated in general population, the CYP2C19*2 loss-of-function allele found by Hulot et al. [4], was found to be associated with weakened antiplatelet response to clopidogrel. However, the findings from another study [5] were not in line with the current investigation, and did not show any influence of the CYP2C19 genotype on the antiplatelet effect after loading with 600 mg clopidogrel in patients with coronary artery disease. Bhatt et al. [6] demonstrated no relationship between CYP2C19 genotype and ischemic events with clinically evident cardiovascular disease or multiple risk factors in patients treated with clopidogrel. Thus, the clinical impact of the CYP2C19 681G > A mutation remains unclear, which requires further study.

In this study, we investigated whether CYP2C19 681G > A mutation is correlated with low inhibition of platelet aggregation (IPA) in ACS patients after administration of clopidogrel. Furthermore, we assessed the correlation between CR at pre-discharge and 12-month incidence of adverse cardiovascular events.

## Patients and Methods

### Study participants

This study was a prospective, single center research. Enrollment began in January 2010, which lasted for two years. Clinical follow-up was completed in January 2013. In this study ACS patients (18–80 years), who underwent percutaneous coronary intervention (PCI), were enrolled. The clinical diagnosis of ACS was based on the 2007 ACC/AHA guideline for the diagnosis and treatment of ACS, including unstable angina (UA), acute ST-segment elevation myocardial infarction (STEMI), and acute non-ST-segment elevation myocardial infarction (NSTEMI). Exclusion criteria included allergic or intolerance to aspirin or clopidogrel, bleeding disorder or tendency, chronic oral anticoagulation drugs (such as warfarin), exposure to clopidogrel in the last 2 weeks, contraindication to antiplatelet therapy, surgery within the preceding 8 weeks, severe anemia (hemoglobin < 60 g/L), renal impairment (serum creatinine > 220 μmol/L), NYHA function class IV, and tumor or severe immune system disorder. This study was approved by the review board of The First Affiliated Hospital of Wannan Medical College. Written informed consent was obtained from all the patients.

The patients were pre-treated with a loading dose of 300 mg clopidogrel and 300 mg aspirin before undergoing coronary angiography, which was followed by the daily administration of 75 mg clopidogrel and 100 mg aspirin. Tirofiban, a glycoprotein IIb/IIIa inhibitor, was administered only when the coronary flow to Thrombolysis In Myocardial Infarction (TIMI) flow grade was < 2.

### Platelet function assays

Blood specimens were collected for platelet function assays using test tubes containing 3.8% sodium citrate before and 24 h after administration of clopidogrel. Samples were processed within 3 hours after blood collection.

Platelet-rich plasma (PRP) was prepared by centrifugation of citrated venous blood at 1000 rpm for 10 min. Residual blood samples, after removing PRP, were centrifuged at 3000 rpm for 5 min and were called as platelet-poor plasma (PPP; 10–20×10^9^ thrombocytes/l). Platelet aggregation was measured using turbidimetric aggregometry by applying a 4-channel LBY-NJ4 aggregometer (Precil, Beijing, China). ADP (Sigma, California, U.S.A.) was used to decrease the aggregation. Light transmission in PRP was measured 5 min after the addition of ADP at a final concentration of 5 μmol/L. Results were expressed as percentage of maximal light transmission using PPP from the same patient as reference (100% aggregation).

Maximum platelet aggregation rate (MPAR) at pre-and-post treatment was determined, and IPA, which was referred to as ΔA (ΔA = pre-treatment PAR-post-treatment PAR), was calculated. ΔA < 10% (including negative value) is referred to as the presence of CR [7]. According to whether or not the value of ΔA < 10%, the ACS patients were classified into CR and non-CR groups.

### Extraction of genomic DNA

Genomic DNA from whole-blood samples was extracted using a purifier (Promega, Wisconsin, U.S.A.) according to the reagent kit instructions.

### Detection of CYP2C19*2 genetic polymorphism by restriction fragment length polymorphism

CYP2C19*2 genetic polymorphism was detected by polymerase chain reaction (PCR)-restriction fragment length polymorphism (RFLP). The sequences were amplified using PCR in a Gradient PCR Thermal Cycler (Labnet, New Jersey, U.S.A.) with conditions listed below: pre-denaturation at 94°C for 5 min, 35 cycles with denaturation at 94°C for 30 sec, annealing at 56°C for 30 sec, extension at 72°C for 20 sec, and final extension at 72°C for 5 min.

To detect the CYP2C19*2 polymorphism, 10 μL of the PCR products (334 bp) were digested with SmaI restriction enzyme (Takara, Dalian, China) and the products were transferred to a water bath (30°C) for 6 h. Agarose gels electrophoresis was performed for the digested fragments (S1 and S2 Figs).

### Follow up

Study visits were conducted for 12 months. The main endpoint was the cumulative incidence of recurrent angina, nonfatal MI, cerebral stroke, stent thrombosis, and sudden cardiac death (SCD). The patients were either directly consulted or contacted by telephone by two referring physicians.

### Statistical analysis

Hardy-Weinberg genetic equilibrium test was used to test the population representativeness. Continuous data was described as mean±SD, whereas the categorical data was represented as counts and percentage. The comparison between the groups was made by *t*-test and one way analysis of variance (ANOVA). Chi square analysis was performed to evaluate the categorical data. Statistical analysis was performed using STATA software version 11.0 (StataCorp.). A *p* value of < 0.05 was considered as statistically significant.

## Results

**Fig 1:**
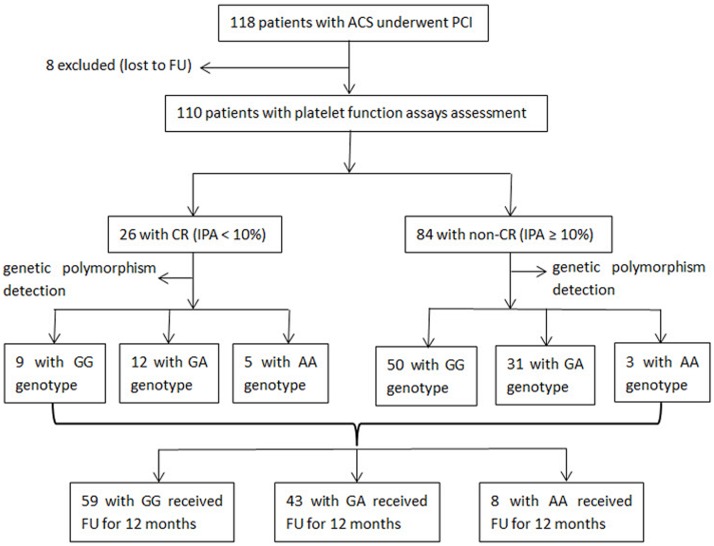
Flowchart of study participants. Flowchart of study participants from enrollment to follow-up. Abbreviations: ACS, acute coronary syndrome; PCI, percutaneous coronary intervention; FU, follow-up; CR, clopidogrel resistance; IPA, inhibition of platelet aggregation.

One hundred eighteen patients (8 patients lost to follow-up) were enrolled for the study (Fig 1). Baseline characteristics of the patients are demonstrated in Table 1. The number of patients with laboratory CR (ΔA < 10%) and non-CR (ΔA ≥ 10%) was 26 (23.6%) and 84 (76.4%), respectively. The demographic, clinical, and laboratory findings for the patients in the groups were similar (Table 1).

**Table 1:** Patient demographics and baseline characteristics.

Characteristic	CR (n = 26)	non-CR (n = 84)	Total (n = 110)	*P* value
**Age (years)**	63.3 ± 11.8	66.0 ± 10.3	65.7 ± 11.7	0.257
**Male sex**	20(76.9)	51(60.7)	71(64.5)	0.880
**Body mass index (kg/m^2^)**	23.5 ± 2.8	23.0 ± 2.8	23.2 ± 2.9	0.447
**Smoking**	12(46.2)	41(48.8)	53(48.2)	0.813
**Hypertension**	14(53.8)	45(53.6)	59(53.6)	0.980
**Diabetes mellitus**	8(30.8)	23(27.4)	31(28.2)	0.737
**Dyslipidaemia**	12(46.2)	47(56.0)	59(53.6)	0.381
**Family history of CAD**	5(19.2)	15(17.9)	20(18.2)	0.874
**UA**	17(65.4)	62(73.8)	79(71.8)	0.404
**STEMI**	7(26.9)	14(16.7)	21(19.1)	0.389
**NSTEMI**	2(7.7)	8(9.5)	10(9.1)	0.777
**β-blockers**	7(26.9)	23(27.4)	30(27.3)	0.963
**ACEIs or ARBs**	18(69.2)	49(58.3)	67(60.9)	0.320
**CCBs**	10(38.5)	27(32.1)	37(33.6)	0.551
**Any statin**	17(65.4)	54(64.3)	71(64.5)	0.918
**Diuretics**	4(15.4)	10(11.9)	14(12.7))	0.642
**Omeprazole**	7(26.9)	20(23.8)	27(24.5)	0.747
**Nitrates**	19(73.1)	41(48.8)	60(54.5)	0.690
**Tirofiban**	8(30.8)	23(27.4)	31(28.2)	0.737

Abbreviations: CR, clopidogrel resistance; CAD, coronary artery disease; UA, unstable angina; STEMI, ST segment elevation myocardial infarction; NSTEMI, non-ST segment elevation myocardial infarction; ACEI, angiotensin-converting enzyme inhibitor; ARB, angiotensin receptor blocker; CCB, calcium channel blocker. Data are expressed as mean ± SD or number of patients (percentage). The p values were determined by 1-way analysis of variance or chi-square between patients with CR and non-CR.

Of the total (110) enrolled patients, 59 patients (53.6%) were CYP2C19 681 wild-type homozygotes (GG), 43 were (39.1%) CYP2C19 681 mutant heterozygotes (GA), and 8 were (7.3%) mutant homozygotes (AA). The IPA was significantly higher in GG genotype group than GA genotype and AA genotype groups (16.2 ± 10.1%, 10.2 ± 9.9%, 8.0 ± 5.9% respectively, *p* < 0.01). After 24 h of clopidogrel administration, the patients in GG group exhibited significantly lower laboratory CR than patients with CYP2C19 681 allele group (9/59 vs. (12+5)/51, *p* = 0.009) (Table 2). Since the number of cases with AA genotype was less, the carriers of CYP2C19 allele in the study cohort were pooled for further analyses.

**Table 2:** Comparison of laboratory clopidogrel resistance between the different CYP2C19 681 genotypes (χ^2^, n = 110).

Genotype	CR	Non-CR	Total
**GG**	9	50	59
**GA**	12	31	43
**AA**	5	3	8
**Total**	26	84	110
**Test results**		χ^2^ = 9.426	*P* = 0.009

Abbreviations as in Table 1.

**Fig 2:**
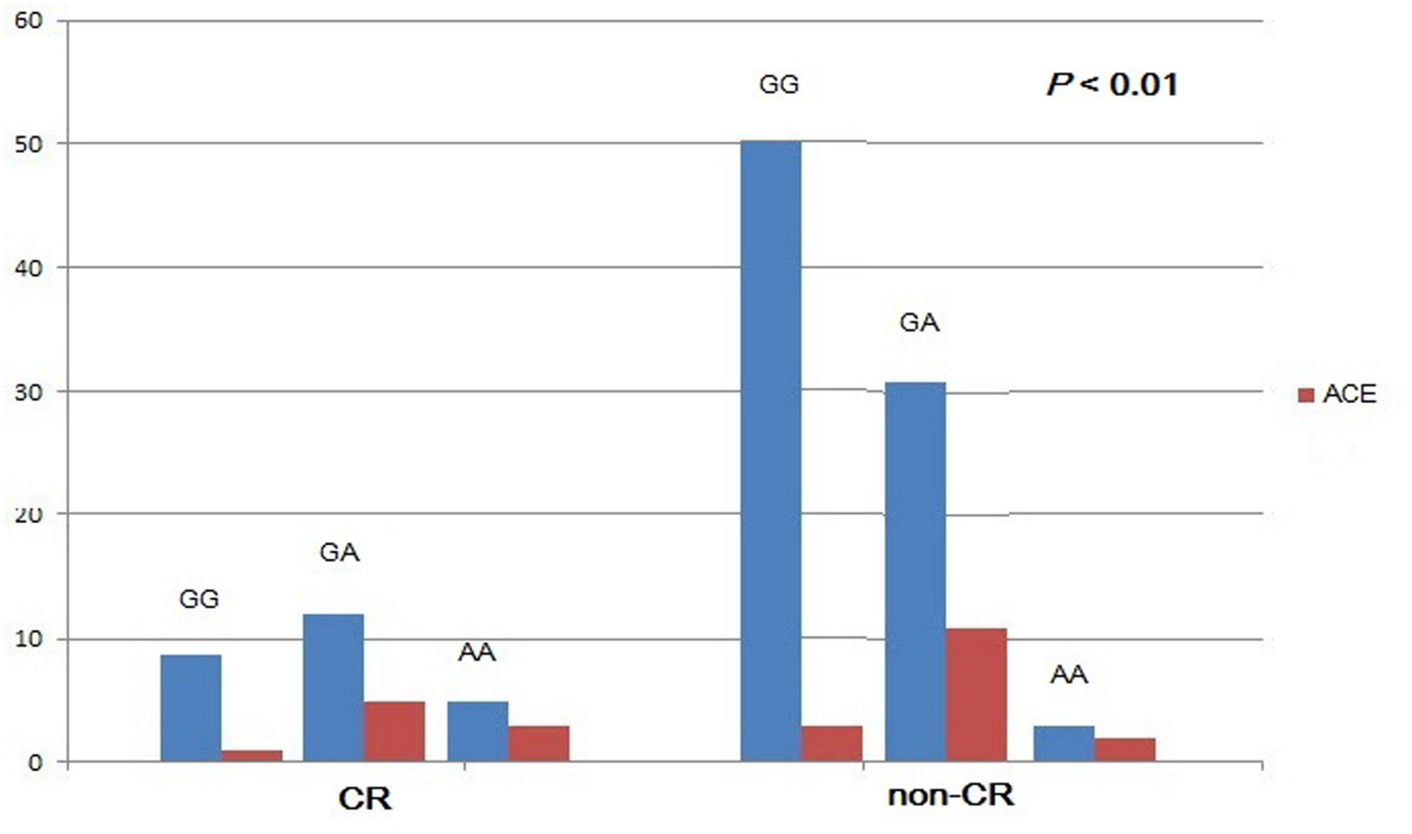
Clinical outcomes in different CYP2C19 genotypes, with or without CR, at 12-month follow-up. Abbreviations: CR, clopidogrel resistance; ACE, adverse cardiovascular events.

The primary clinical endpoint, which was the cumulative incidence of recurrent angina, AMI, cerebral stroke, intra-stent thrombosis, and SCD, was observed in 25 patients (22.7%) during the follow-up. Recurrent angina, AMI, and intra-stent thrombosis occurred less often in CYP2C19 681 GG patients than CYP2C19 681A allele (GA+AA) patients (2/59 vs. 8/51, 1/59 vs. 6/51, 0 vs. 4/51, respectively, *p* < 0.05). However, the incidence of cerebral stroke and SCD was similar in both the groups (*p* > 0.05) (Table 3). The clinical outcomes in different CYP2C19 genotypes, with or without CR, at 12 months follow-up are shown in Fig 2.

**Table 3:** Occurrence of adverse cardiovascular events between the different CYP2C19 681 genotypes.

	GG (n = 59)	GA + AA (n = 51)	P value
**Recurrent angina**	2	8 (7+1)	0.025
**AMI**	1	6 (4+2)	0.031
**Stent thrombosis**	0	4 (3+1)	0.028
**Cerebral stroke**	1	1 (0+1)	0.917
**SCD**	0	2 (2+0)	0.125

Abbreviations: AMI, acute myocardial infarction; SCD, sudden cardiac death. Stent thrombosis was defined according to Academic Research Consortium criteria. Data are expressed as number of patients.

## Discussion

Cytochrome P450 (CYP450) enzymes belong to a superfamily of heme-containing monooxygenases [8]. The function of CYP450 in disease predisposition and drug metabolism has been well researched. Polymorphisms in CYP450 genes affect the expression and/or activity of the enzyme, which leads to adverse clinical outcomes. So far, 57 active CYP450 enzymes have been identified in humans [9]. The CYP2C sub-family (CYP2C8, CYP2C9, CYP2C18, and CYP2C19) is responsible for the metabolism of approximately 20% of all CYP450 substrates [10]. CYP2C8, CYP2C9, and CYP2C19 are the most pharmacologically active enzymes of this sub-family. CYP2C19 also carries out the transformation of the prodrug, clopidogrel into an active metabolite. Several studies have demonstrated the correlation between the variants in the CYP2C19 gene and the risk of adverse cardiovascular outcomes in clopidogrel-treated patients [11–13]. In patients with coronary artery disease carrying the genetic variant associated with a loss of function of the CYP2C19 enzyme, the risk for stent thrombosis is 3–6 times higher with clopidogrel treatment [10–12]. However, the effect and risk stratification associated with CYP2C19 genotype are controversial among different studies. A genome wide association study demonstrated that the polymorphic hepatic CYP2C19 enzyme accounts for only 12% of the variation in response to clopidogrel [14]. Other genetic and environmental factors may also play a critical role, which require further analysis. The present studies showed that 681G > A mutation (CYP2C19*2) in CYP2C19 gene polymorphism loci is closely related to individual differences in the efficacy of clopidogrel [15–18].

Our data suggested that the presence of CYP2C19*2 polymorphism is associated with higher laboratory CR in ACS patients (especially those who underwent PCI). We found that the carriers of CYP2C19 681 allele are associated with 3-fold increased risk for adverse cardiovascular events when treated with clopidogrel. From these findings, it was inferred that that patients with CYP2C19*2 allele treated with clopidogrel post-PCI should be warned about the risk of these clinical events. It is necessary to recognize the CYP2C19*2 loss of function in patients with ACS undergoing PCI, including relevant clinical features of weakened functional reaction to clopidogrel. Increasing the dose of clopidogrel may be one of the options to overcome CR in ACS patients with low- or non-response to clopidogrel [19].

## Conclusion

CYP2C19 681 G > A mutation, associated with reduced clopidogrel antiplatelet activity, is an important marker for poor prognosis of ACS patients receiving clopidogrel treatment. Genotyping of CYP2C19*2 has the potential to guide the use of antiplatelet treatment.

## Limitations

The sample size was relatively small, and the results could not be extrapolated to all the ACS population. In order to validate the results further, the clinical trials involving much larger patient cohorts are required.

## Supporting Information

S1 Fig The electrophoretic patterns of the amplified products of different CYP2C19 681 genotypes. (TIF)

S2 Fig The sequencing result of different CYP2C19 681 genotypes. A: GG genotype; B: GA genotype; B: AA genotype.
(TIF)
