# Supplementary figures and images for: Cytochrome P450 CYP 2C19*2 Associated with Adverse 1-Year Cardiovascular Events in Patients with Acute Coronary Syndrome
Source: PLoS One. 2015 Jul 6;10(7):e0132561. doi: 10.1371/journal.pone.0132561 (PMC4493116; doi:10.1371/journal.pone.0132561)

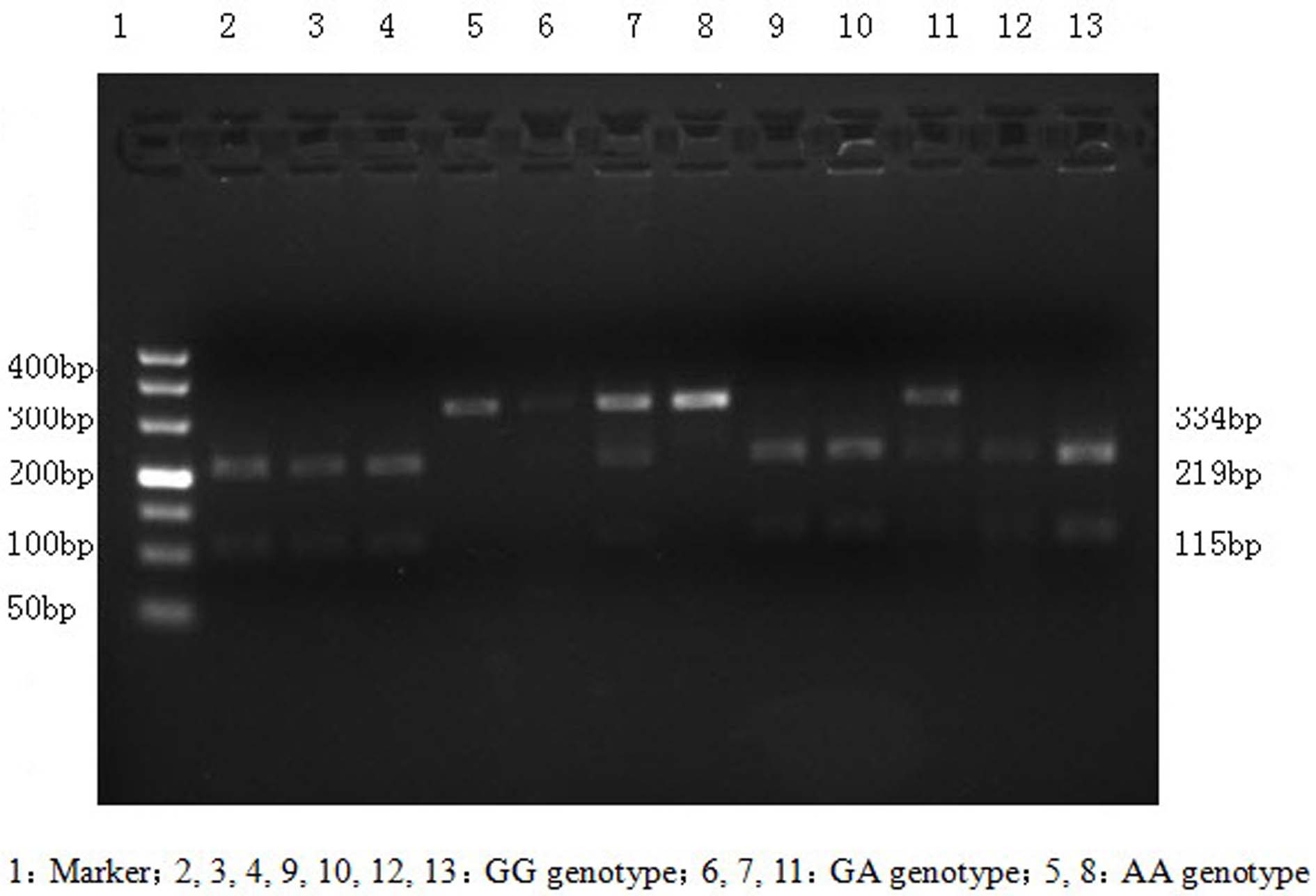

Supplement: S1 Fig — (TIF) [file pone.0132561.s001.tif]

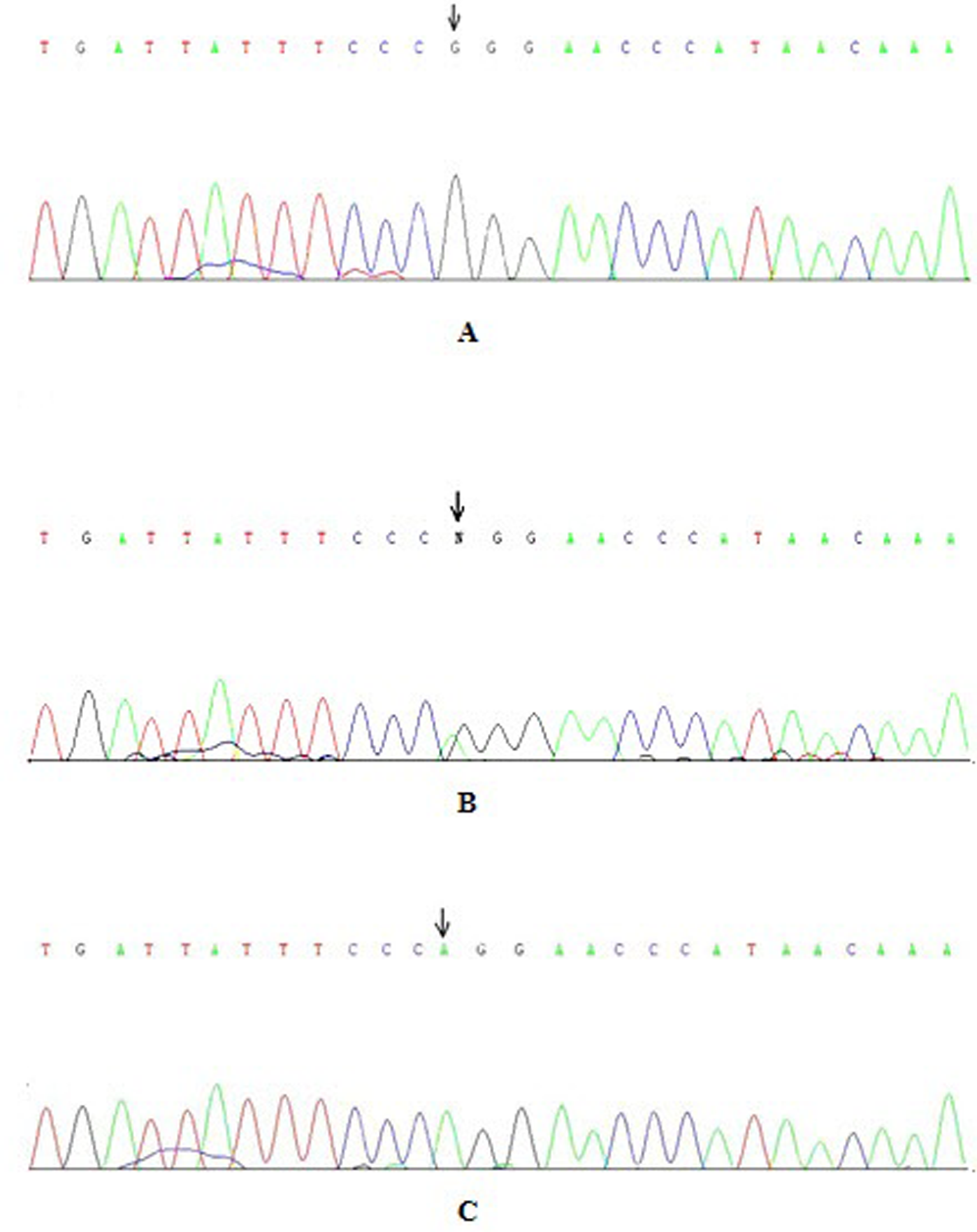

Supplement: S2 Fig — A: GG genotype; B: GA genotype; B: AA genotype. (TIF) [file pone.0132561.s002.tif]
